# Flavonoid-Rich Extracts from Chuju (*Asteraceae Chrysanthemum* L.) Alleviate the Disturbance of Glycolipid Metabolism on Type 2 Diabetic Mice via Modulating the Gut Microbiota

**DOI:** 10.3390/foods14050765

**Published:** 2025-02-24

**Authors:** Yu Yin, Wen Nie, Zheng-Quan Tang, Shuang-Jie Zhu

**Affiliations:** 1 School of Life Sciences, Anhui University, Hefei 230601, China; 15556918670@163.com; 2 School of Biological and Food Engineering, Chuzhou University, Chuzhou 239001, China; niewen@chzu.edu.cn

**Keywords:** chuju extract, type 2 diabetes, glycolipid metabolism, gut microbiota

## Abstract

Type 2 diabetes mellitus (T2DM) and its associated complications represent a significant public health issue affecting hundreds of millions of people globally; thus, measures to prevent T2DM are urgently needed. Chuju has been proven to possess antihyperglycemic activity. However, the bioactive ingredients in chuju that contribute to its antihyperglycemic activity, as well as the relationship between its antihyperglycemic activity and the gut microbiota, remain unclear. To understand the potential effects that it has on T2DM, the glycolipid metabolism and gut microbiota regulation of flavonoid-rich extracts from chuju (CJE) were investigated. The results showed that the top ten flavonoid compounds in CJE are Apigenin 6, 8-digalactoside, Apigenin 6-C-glucoside 8-C-arabinoside, Luteolin-4′-O-glucoside, Isoshaftoside, Scutellarin, Quercetin 3-O-malonylglucoside, Chrysoeriol 7-O-glucoside, Quercetin-3,4′-O-di-beta-glucoside, Luteolin 6-C-glucoside 8-C-arabinoside, and Homoorientin. Furthermore, CJE mitigated hyperglycemia and glycolipid metabolism by reducing the abundance of *Faecalibaculum*, *Coriobacteriaceae*, and *Romboutsia* and increasing the abundance of *Alistipes*. In addition, the results of Western blot analysis showed that CJE could enhance glycogen synthesis and glucose transport by up-regulating the phosphorylation of IRS1-PI3K-Akt and AMPK-GLUT4. Simultaneously, CJE could decrease gluconeogenesis by down-regulating the phosphorylation of FoxO1/GSK 3β. In conclusion, the findings of this study provide new evidence supporting the hypothesis that CJE can be used as part of a therapeutic approach for treating disturbances in glycolipid metabolism via regulating the gut microbiota and mediating the IRS1-PI3K-Akt-FoxO1/GSK 3β and AMPK-GLUT4 pathways.

## 1. Introduction

Diabetes mellitus, the most common form of chronic metabolic disorder, is characterized by persistent hyperglycemia, insulin resistance, and abnormal disturbances in glycolipid metabolism [1]. According to the latest epidemiological research, the global prevalence of diabetes has risen dramatically in recent years and will continue to increase to 12.2% (783.2 million people) by 2045 [2], with nearly 90% of diabetics being diagnosed with type 2 diabetes mellitus (T2DM) [3]. A persistent disturbance in glycolipid metabolism can lead to progressive lesions and even failure in multiple organs, including the eyes, kidneys, heart, and blood vessels [4]. Currently, the main strategies for the treatment of T2DM are a carbohydrate-restricted diet and the administration of hypoglycemic drugs, such as sulfonylureas, biguanides, and lignans [5]. However, these medications have functional limitations and may contribute to gastrointestinal disturbances when taken over a long period of time [6,7,8]. In recent years, the search for safe and nontoxic bioactive substances from natural sources to prevent and ameliorate T2DM has attracted the attention of more and more food nutritionists.

The gut microbiota refers to the more than 10^14^ bacteria in the human intestine that participate in glucolipid metabolism [9], neurological function [10], and the immune response [11] for maintaining human health. The relationship between gut microbiota and T2DM is complex and multifactorial [12,13]. Consequently, regulating the homeostasis of the intestinal flora should be regarded as a possible strategy for treating T2DM.

Chuju (*Asteraceae Chrysanthemum* L.) is a plant of the composite family and is known as one of the four medicinal chrysanthemums, and it has a long history of being used in traditional medicine and tea in China [14]. Chuju is recognized as a plant with high nutritional value as well as anti-inflammatory [15], antioxidant [16], and hepatoprotective effects [17], containing various phytochemical substances, including polysaccharides, flavonoids, phenolic acids, volatile oils, and other natural active ingredients [18]. Additionally, flavonoids from chuju have been demonstrated to be beneficial in regulating the level of blood glucose [19]. However, the effect of flavonoid extracts from chuju on the gut microbiota and the potential mechanism of alleviating disturbances in glycolipid metabolism remain unclear.

The aim of this study was to evaluate the effect of flavonoid extracts from chuju on disturbances in glycolipid metabolism using a mouse model of type 2 diabetes, mediated via a high-fat diet (HFD) with streptozotocin (STZ). Furthermore, this study investigates the role of the gut microbiota in mediating the effects of chuju on glycolipid metabolism. This study aims to provide insights into the potential of flavonoid-rich extracts of chuju as a natural therapeutic strategy for T2DM.

## 2. Materials and Methods

### 2.1. Materials and Chemicals

Chuju was purchased from Anhui Jutai Chuju herbal technology Co. (Chuzhou City, China), LTD. AB-8 macroporous resin, recombinant human insulin, rutin standards, streptozotocin (STZ), metformin hydrochloride, and sodium citrate buffer were purchased from Beijing Solarbio Science & Technology Co., Ltd. (Beijing, China). D-(+)-Glucose, sucrose, and anhydrous ethanol were obtained from Sigma-Aldrich (St. Louis, MO, USA). The primary antibodies IRS-1, phosphor-Akt (p-Akt) (Ser473), phosphor-FoxO1 (p-FoxO1) (Ser256), phosphor-GSK-3β (p-GSK3β) (Ser9), and β-actin were obtained from Wuhan service biotechnology Co., Ltd. (Wuhan, China). AMPKα, phosphor-AMPKα (p-AMPK) (Thr172), FoxO1, and GLUT4 were purchased from Affinity (Cincinnati, OH, USA). Phosphor-IRS-1 (p-IRS-1) (Ser307), PI3K, phosphor-PI3K (p-PI3K) (Tyr467/199), Akt, and GSK-3β were obtained from Abmart (Shanghai, China).

### 2.2. Preparation of Flavonoids from Chuju

CJE was extracted by the method of ultrasonic-assisted ethanol solvent extraction according to the study by Huang et al. with minor modifications to the extraction temperature and time [20]. Freshly dried chuju was pulverized and sieved to a particle size of less than 100 mesh. Then, 100 g of chuju powder and 3100 mL of ethanol solution (77% *v*/*v*) were mixed and extracted with an ultrasonic extractor at 200 W for 25 min at 45 °C (GS-100A multi-function ultrasonic machine, Shenzhen, China). Subsequently, the homogenate was centrifuged at 6000 rpm for 20 min at 4 °C. An equal volume of anhydrous ethanol was added to the supernatant, and the mixture was stored in a refrigerator for 12 h at 4 °C to precipitate carbohydrates and proteins in the supernatant. Then, the supernatant was dried in a vacuum freeze dryer to obtain the flavonoid extract of chuju. The obtained extract of flavonoids (500 mg/mL) was subjected to a chromatography column (2.5 × 100 cm) packed with AB-8 macroporous resin (M0042) to fractionate the different components. The separation was performed at a flow rate of 2.0 mL/min with 35% (*v*/*v*) ethanol solution at room temperature. The separated components were assayed using an ultraviolet detector at 280 nm. Finally, the separated components were dried in a vacuum freeze dryer for further analysis.

The compositions of CJE were measured using an ultra-high-pressure liquid chromatography system equipped with a 6600 QTOF detector (UPLC-QTOF) (Waters Corporation, Milford, MA, USA) with an ACQUITY UPLC HSS T3 column (1.8 μm, 2.1 × 100 mm). The UPLC-QTOF conditions were based on a previous study [21]. The main components of flavonoids were Apigenin 6, 8-digalactoside, Apigenin 6-C-glucoside 8-C-arabinoside, Luteolin-4′-O-glucoside, Isoshaftoside, Quercetin 3-O-malonylglucoside, Chrysoeriol 7-O-glucoside, Scutellarin, Quercetin-3,4′-O-di-beta-glucoside, Luteolin 6-C-glucoside 8-C-arabinoside, and Homoorientin (Table 1). The content of total flavonoids in CJE was 78.57 ± 5.18%. Simultaneously, the remaining components mainly consisted of protein (5.48 ± 1.03%) and sugar (4.22 ± 0.94%).

### 2.3. Animal Experiments

Seventy male C57BL/6 mice (4 weeks old, 18–20 g) with the animal certificate number SCXK (Jin) 2022-006 were obtained from the SPF Biotechnology Co., Ltd. (Beijing, China). The mice were adaptively fed for one week at 25 ± 2 °C and 60 ± 5% relative humidity with a 12 h light–dark cycle and free access to water and food. All the experiments were approved by the biomedical ethics committee of Anhui University and were carried out in strict accordance with the relevant guidelines.

Excluding one week of adaptation, the mice were randomly divided into two groups: one fed a basal diet (*n* = 20) and one fed a high-fat diet (D12492, Xietong Pharmaceutical Bio-engineering Co., Ltd., Nanjing, China) (*n* = 50). After 15 weeks, the mice on the high-fat diet were given STZ (dissolved in 0.1 mol/L citrate buffer, pH 4.5) once at a dose of 100 mg/kg body weight (BW) via intraperitoneal injection after fasting overnight. By contrast, the mice on the basal diet were given the same volume of citrate buffer (0.1 mol/L, pH 4.5) via intraperitoneal injection. Then, the fasting blood glucose (FBG) levels of mice on the high-fat diet were measured using Accu-Chek glucometers (Roche Diagnostics, Mannheim, Germany) after one week. A level of FBG greater than 11.1 mmol/L in two consecutive analyses was considered to indicate the successful construction of the T2DM mouse models [22]. Subsequently, the mice on the basal diet were randomized into two groups: the normal control group (NC, *n* = 10) and the normal mice with the CJE group (NC + CJE, 400 mg/kg/day, *n* = 10). The T2DM mice were randomly divided into five groups (*n* = 10): the positive control group (PM, metformin, 200 mg/kg/day), the diabetic control group (DC), the diabetic with low-dose CJE group (CL, 100 mg/kg/day), the diabetic with medium-dose CJE group (CM, 200 mg/kg/day), and the diabetic with high-dose CJE group (CH, 400 mg/kg/day). The normal control group and the diabetic control group were orally gavaged an equal amount of normal saline once daily for an additional 6 weeks. CJE and metformin were dissolved in sterile saline. During the whole experiment, the normal control group and the normal mice with the CJE group were fed a basal diet, while the others were fed a high-fat diet. The levels of FBG, food intake, water consumption, and body weight of each group were monitored weekly. After the last week of the experiment, all mice fasted overnight. Finally, all mice were euthanized in a CO_2_ chamber, followed by the collection of blood, liver, colon, pancreas, and white adipose tissue (WAT) (including epididymal fat, perirenal fat, and mesenteric fat) for further analysis.

### 2.4. Oral Glucose and Insulin Tolerance Tests

The oral glucose tolerance test (OGTT) was investigated according to a previously described method with minor modifications [23]. After 6 weeks of the CJE treatment by gavage, the OGTT was performed in all mice from the normal control group, normal mice with the CJE group, diabetic control group, positive group, and diabetic with the CJE group. All mice were orally administrated 2 g/kg BW glucose after being deprived of food for 12 h, while water was still provided ad libitum. The levels of blood glucose were determined at different time points after glucose administration in different time periods (0, 15, 30, 60, 90, and 120 min) using Accu-Chek glucometers.

The insulin tolerance test (ITT) was evaluated according to a previous study with minor modifications [24]. Mice were intraperitoneally injected with human recombinant insulin (0.75 unit/kg BW), and the blood was taken from the tail tip to measure the concentration of blood glucose using Accu-Chek glucometers at 0, 15, 30, 60, 90, and 120 min post-injection. To mitigate stress-induced hyperglycemia, all mice had their tails pinched for a week in order for them to acclimatize prior to the OGTT and ITT [25].

### 2.5. Biochemical Parameter Analysis

Serum was collected after 3500 rpm centrifugation for 20 min at 4 °C. The levels of glycogen, fasting serum insulin (FINS), glycosylated serum protein (GSP), total triglyceride (TG), total cholesterol (TC), low-density lipoprotein cholesterol (LDL-C), and high-density lipoprotein cholesterol (HDL-C) were assayed using commercially available kits from Nanjing Jiancheng Bioengineering Institute (Nanjing, China). The homeostasis model assessment of insulin resistance (HOMA-IR), β-cell function (HOMA-β), and the quantitative insulin sensitivity check index (QUICKI) were calculated according to the following formulas, respectively [26]:

HOMA-IR = FBG (mmol/L) × FINS (mIU/L)/22.5

HOMA-β = 20 × FINS (mIU/L)/[FBG (mmol/L) − 3.5]

QUICKI = Ln[FINS (mIU/L) × FBG (mmol/L)]^−1^

### 2.6. Histological Assessment

The liver, pancreas, and colon tissues were fixed with 4% paraformaldehyde solution at 4 °C for more than 24 h. Then, they were embedded in paraffin and sectioned into 5 μm slices using a freezing microtome (CRYOSTAR NX50, Thermo Fisher Scientific, Shanghai, China). The liver, pancreas, and colon tissues were stained with hematoxylin–eosin (H&E) (hematoxylin dye for 5 min, washed with distilled water, returned to the blue solution, and washed again; then, the sections were dehydrated with 95% alcohol for 5 min and then stained with eosin solution for 15 s) to observe histopathological morphology. Periodic Acid–Schiff (PAS) (PAS solution B dyed for 15 min, rinsed thrice with distilled water, dyed with PAS solution A for 25 min in the dark, washed with distilled water, and then dyed with PAS solution C for 30 s, washed, and treated the slices with hydrochloric acid solution and ammonia) was used to measure liver glycogen levels and with Oil Red O (oil red O solution dye for 10 min in the dark, 60% isopropyl alcohol for differentiation, washed with distilled water, and dyed with hematoxylin for 5 min) to observe the condition of lipid accumulation. All images were scanned using a Pannoramic DESK (3DHISTECH) (3DHISTECH Ltd., Budapest, Hungary). The quantification of the islet area and total hepatic glycogen area was conducted using Image J software 1.54g (National Institutes of Health, Bethesda, MD, USA).

### 2.7. Analysis of Gut Microbiota

DNA of the gut microbiota was extracted from the cecal contents according to the instructions of the Omega EZNATM fecal DNA extraction kit (Omega Bio-tek, Norcross, GA, USA). The concentration of DNA in the extract was quantitatively analyzed using a NanoDrop 1000 nucleic acid concentration analyzer (Thermo Scientific, Wilmington, NC, USA), and the quality of DNA was qualitatively analyzed via electrophoresis on 1% agarose gels. The samples meeting the sequencing requirements (a DNA concentration greater than 50 ng/μL and clear bands) were stored in a refrigerator at −80 °C. To minimize the effects of sequencing depth on alpha and beta diversity measure, 16S rRNA gene sequences from each sample were rarefied to 44283. Thermal cycle PCR (GeneAmp 9700, Applied Biosystems Inc., Foster City, CA, USA) was used to analyze the structure of the intestinal microbiota based on the V3–V4 variable region of the bacterial 16S rRNA gene. The V3–V4 variable region of the 16S rRNA gene of the bacterial genome was amplified with 338F (5′-ACTCCTACGGGAGGCAGCAGCAG-3′) and 806R (5′-GGACTACHVGGGTWTCTAAT-3′) as primers. The GMHI was calculated according to a previous study [27]. The results were analyzed using the Majorbio cloud platform (https://www.majorbio.com) (accessed on 22 December 2024).

### 2.8. Western Blot Analysis

The liver tissues were thoroughly homogenized with 10 times the volume of a RIPA buffer containing 1% protease inhibitors for 60 s. After adding the loading buffer, all protein samples were denatured in a boiling water bath for 15 min. The supernatant was collected by centrifuging the mixture at 12,000× *g* for 30 min at 4 °C, providing the total protein solution. The protein concentration in the supernatant was determined using the BCA protein concentration assay kit (Wuhan Xavier Biotechnology Co., LTD, Wuhan, China). Aliquots containing 5 µL protein samples were separated via 10% sodium dodecyl sulfate–polyacrylamide gel electrophoresis (SDS-PAGE) at 80 V for 40 min and then upgraded to 120 V. The bands were transferred from the glue onto a polyvinylidene fluoride (PVDF) membrane under the action of a constant current of 300 mA for 55 min. After sealing for 1 h, PVDF membranes were washed with TBST 3 times for 5 min each time. PVDF membranes were performed using the primary monoclonal antibodies against p-IRS1, IRS1, p-PI3K, PI3K, p-Akt, Akt, p-FoxO1, FoxO1, p-GSK 3β, GSK 3β, p-AMPK, AMPK, and GLUT4 (all dilution ratios 1:1000), respectively, and incubated at 4 °C overnight. After washing the membranes with TBST, the HRP-conjugated secondary antibodies were administered at room temperature for 1 h (1:5000). We used anti-rabbit β-actin as an internal reference. The Western blot images were captured with the AIWBwell System (Wuhan servicebio technology Co., Ltd., Wuhan, China) and quantified by densitometric analysis using Image J software 1.54g (National Institutes of Health, Bethesda, MD, USA).

### 2.9. Statistical Analysis

All data were presented as mean ± SEM. Significant differences among all groups were calculated using one-way ANOVA followed by the Bonferroni post hoc test. All statistical analyses were performed using Graph Pad Prism 8.0.2 software (GraphPad Software, La Jolla, CA, USA). *p* values < 0.05 were considered statistically different.

## 3. Results

### 3.1. CJE Ameliorated Basal Physiological Indicators in T2DM Mice

**Figure 1:**
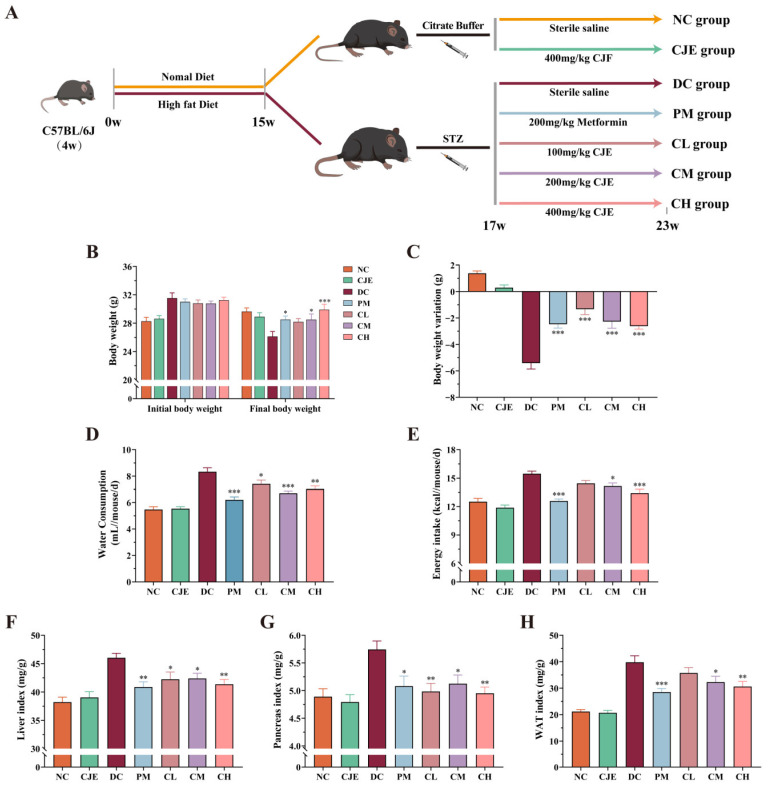
Effects of CJE on the basal physiological indicators in T2DM mice (*n* = 10). (**A**) Treatment of each experimental group. (**B**) Initial (week 17) body weight and final (week 23) body weight. (**C**) Body weight variation over 6 weeks. (**D**) Water consumption at the end of the 6-week administration period. (**E**) Energy intake at the end of the 6-week administration period. (**F**) The liver index. (**G**) The pancreatic index. (**H**) The white adipose tissue (WAT) index. Data are presented as the mean ± SEM. * *p* < 0.05, ** *p* < 0.01, *** *p* < 0.001 vs. the DC group.

**Figure 2:**
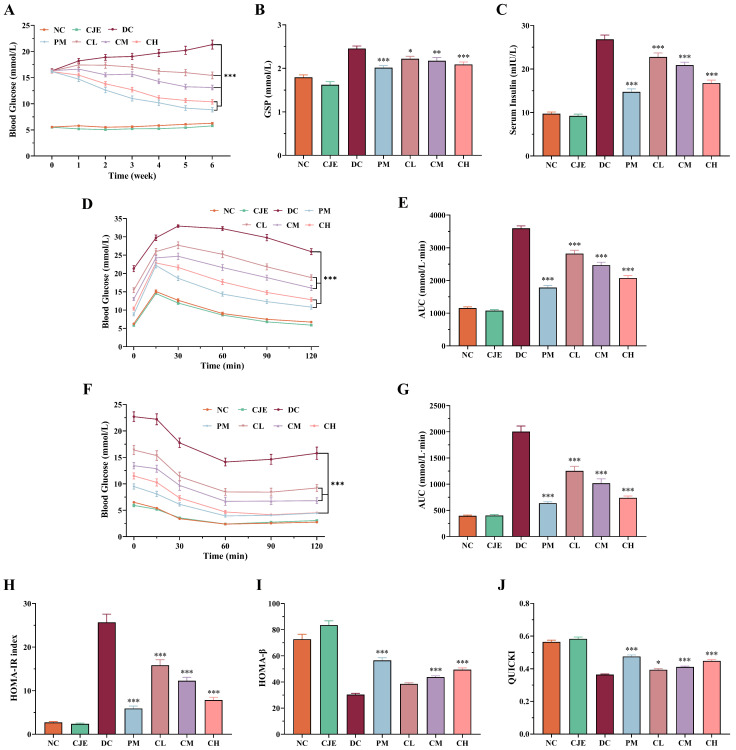
Effect of CJE on blood glucose homeostasis in T2DM mice (*n* = 10). (**A**) FBG levels of each group of mice during 6 weeks (*n* = 10). (**B**) Effect of CJE on glycated serum protein (GSP) level in each group of mice. (**C**) Serum insulin levels at the end of the 6-week administration period. (**D**) Curve of the OGTT. (**E**) AUCs of the OGTT. (**F**) Curve of the ITT. (**G**) AUCs of the ITT. (**H**) HOMA-IR at the end of the 6-week administration period. (**I**) HOMA-β at the end of the 6-week administration. (**J**) Quantitative insulin sensitivity check index (QUICKI) at the end of the 6-week administration period. Data are presented as mean ± SEM. * *p* < 0.05, ** *p* < 0.01, *** *p* < 0.001 vs. the DC group.

The experimental design is shown in Figure 1A. Increases in FBG, food intake, water consumption, and weight loss are generally considered indicators of the occurrence of type 2 diabetes. Compared with the NC group, the level of FBG in the DC group significantly increased and remained above 11.1 mmol/L after pretreatment with a high-fat diet combined with STZ (Figure 2A). These results indicated that the model of diabetic mice was successfully established. Simultaneously, the levels of weight loss, energy intake, and water consumption in diabetic mice with the medium and high doses of the CJE treatment were significantly higher (*p* < 0.05) than those of the DC group (Figure 1B–E). In addition, the basal physiological indicators, including the liver index, pancreas index, and WAT index, were explored in diabetic mice with oral administration of CJE. The results showed that CJE administration could effectively slow down the trend of weight loss and significantly lower the increase in the levels of water consumption, energy intake, liver index, pancreas index, and WAT index in diabetic mice (Figure 1F–H).

### 3.2. CJE Alleviated Hyperglycemia and Insulin Resistance in T2DM Mice

The effects of CJE dosage on the level of FBG in diabetic mice were investigated in this study. As shown in Figure 2A, the level of FBG in normal mice receiving the oral administration of CJE at a dose of 400 mg/kg/day was not significantly different (*p* > 0.05) in comparison to the NC group. In contrast, compared with the DC group, the levels of FBG in the CL, CM, and CH groups were significantly decreased (*p* < 0.05) in a dose-dependent manner. These results indicate that CJE alleviates the disturbance of FBG in type 2 diabetic mice. Moreover, GSP is taken as a key clinical indicator to diagnose the level of glycemic control [28]. As shown in Figure 2B, the level of GSP in the DC group significantly increased (*p* < 0.05) compared with the NC and CJE groups. On the contrary, the levels of GSP in diabetic mice treated with metformin (PM group) and CJE (CL, CM, and CH group) significantly decreased in comparison to the DC group. Moreover, the levels of GSP in the CH group were lower than in the CL and CM groups. This result indicates that CJE could significantly ameliorate the increasing trend regarding the GSP level in diabetic mice in a dose-dependent manner.

The OGTT experiment is commonly used to measure the ability of animals to respond to acute hyperglycemia. As shown in Figure 2D, the blood glucose levels of mice in each group increased dramatically, and the blood glucose levels of mice in the DC, CL, and CM groups reached a peak value at 30 min, while in the NC, CJE, PM, and CH groups, they reached a peak value at 15 min after glucose gavage. Compared to the DC group, the blood glucose levels of mice in the PM, CL, CM, and CH groups were significantly decreased (*p* < 0.05) at the termination of the 120 min OGTT experiment. Simultaneously, the area under the curve (AUC) of the OGTT experiment was significantly decreased in the PM (50.45%), CL (21.50%), CM (31.28%), and CH (42.30%) groups compared with the DC group at the end of the OGTT experiment (Figure 2E). The ITT experiment is commonly used to evaluate the insulin sensitivity of animals. As shown in Figure 2F, the blood glucose levels of mice in the PM, CL, CM, and CH groups were significantly decreased (*p* < 0.05) at the termination of the 120 min ITT experiment. Notably, the CH group was able to achieve the same efficacy as the PM group. Compared with the DC group, the area under the curve (AUC) of the ITT experiment was significantly decreased in the PM (68.24%), CL (37.40%), CM (49.23%), and CH (63.17%) groups at the end of the ITT experiment (Figure 2G). These results show that CJE can significantly enhance glucose tolerance and improve insulin tolerance.

Insulin is a key hormone in animals and it is used to lower blood glucose and is considered an important index to reflect the insulin storage and secretion functions in cells. Compared with the NC group, the level of serum insulin significantly increased (*p* < 0.05) in the DC group. As shown in Figure 2C, the levels of serum insulin significantly decreased (*p* < 0.05) in the PM (45.07%), CL (15.10%), CM (22.14%), and CH (37.52%) groups in comparison to the DC group. The levels of HOMA-IR, HOMA-β, and QUICKI are commonly used to assess islet β-cell function and insulin resistance. Compared with the NC group, the level of HOMA-IR significantly increased (*p* < 0.05) in the DC group. As shown in Figure 2H, the levels of HOMA-IR significantly decreased (*p* < 0.05) in the CL (38.33%), CM (52.21%), and CH (69.44%) groups in comparison to the DC group. Concurrently, the levels of HOMA-β and QUICKI in the DC group significantly decreased (*p* < 0.05) in comparison to the NC group. As illustrated in Figure 2I, the levels of HOMA-β in the CM (44.08%) and CH (62.81%) groups significantly increased (*p* < 0.05) in comparison to the DC group. Compared with the DC group, the level of QUICKI significantly increased (*p* < 0.05) in the CL (8.43%), CM (13.07%), and CH (23.31%) groups (Figure 2J). These results suggest that CJE can effectively improve glucose tolerance and insulin sensitivity, thus ameliorating insulin resistance.

### 3.3. CJE Alleviated the Damage to Pancreatic Morphology and β-Cell Function in T2DM Mice

**Figure 3:**
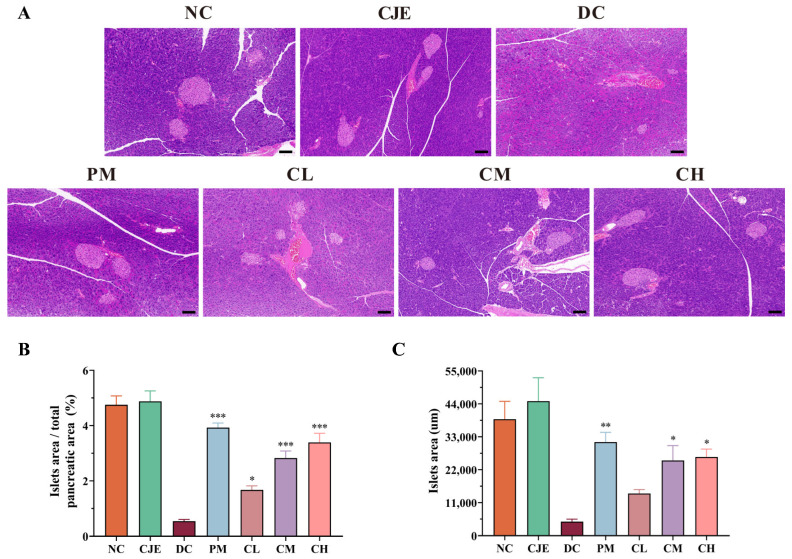
Histological analysis of the pancreas based on H&E staining. (**A**) Pancreas sections of mice. Scale bar, 100 μm. (**B**) Quantification of islet area was calculated based on H&E staining of pancreatic sections. (**C**) Quantification of the area of the largest islets. Data are presented as mean ± SEM. * *p* < 0.05, ** *p* < 0.01, *** *p* < 0.001 vs. the DC group.

The pancreas plays an important role in the control of blood glucose by secreting insulin and glucagon [29]. Compared with the NC group, the pathological structure of the pancreas displayed the unorganized distribution of histiocytes accompanied by focal necrosis and lymphocytic infiltration (Figure 3A). This result indicates that the CJE treatment could significantly improve pancreatic injury in diabetic mice. Pancreatic β-cells belong to the endocrine part of the pancreas, which is responsible for the secretion of insulin to maintain the body’s blood glucose levels and is closely related to the occurrence of type 2 diabetes [30]. The pancreatic β-cells of normal mice were large, plump, and regular in shape with clear edges. Meanwhile, the pancreatic β-cells showed atrophy and apoptosis with significant reductions in volume and number and irregular cell morphology accompanied by blurred area margins in T2DM mice. Compared with the DC group, the ratio of the β-cell area to the pancreatic area increased in a dose-dependent manner after GJE intervention (Figure 3B). The area of the largest beta cells in the pancreas was significantly increased after medium and high doses of CJE (Figure 3C). These results suggest that CJE could alleviate the disturbances in glucose metabolism in T2DM mice by improving the pathological structure of the pancreas and promoting the regeneration of pancreatic β-cells.

### 3.4. CJE Regulated Lipid Profile Disturbances and Ameliorated Liver Injury in T2DM Mice

**Figure 4:**
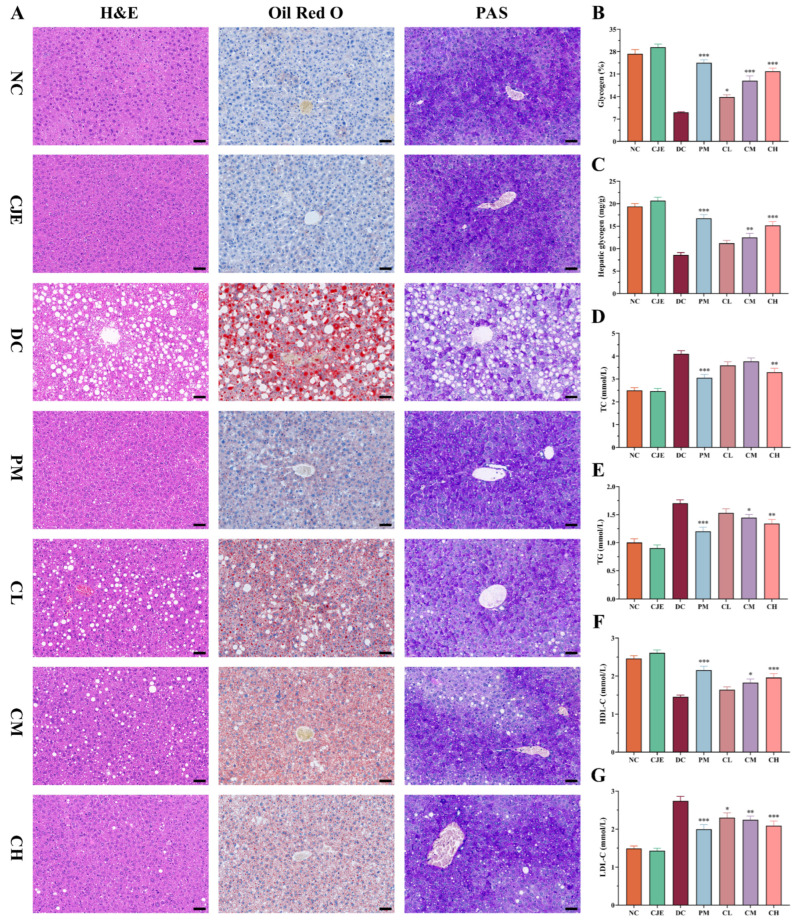
Effects of CJE administration on pathological features and lipid and glucose metabolism in the liver in T2DM mice (*n* = 10). (**A**) Representative images from sections stained with H&E, PAS, and Oil Red O. Scale bar, 50 μm. (**B**) Quantification of hepatic glycogen in PAS-stained sections. (**C**) Effect of CJE on glycogen content in the liver tissue. (**D**) Total cholesterol (TC) levels. (**E**) Triglyceride (TG) levels. (**F**) High-density lipoprotein cholesterol (HDL-C) levels. (**G**) Low-density lipoprotein cholesterol (LDL-C) levels. The data are expressed as the mean ± SEM. * *p* < 0.05, ** *p* < 0.01, *** *p* < 0.001 vs. the DC group.

The liver is one of the important organs for the regulation of glucolipid metabolism, playing an important role in the pathogenesis of diabetes [31]. As shown in Figure 4D–G, the levels of TC (4.1 mmol/L), TG (1.7 mmol/L), and LDL-C (2.74 mmol/L) in the DC group were significantly higher (*p* < 0.05) in comparison to the NC group. Meanwhile, the levels of HDL-C (1.45 mmol/L) significantly decreased in the DC group. Fortunately, the levels of TC, TG, and LDL-C significantly decreased (*p* < 0.05) in diabetic mice receiving moderate and high doses of CJE. Simultaneously, the decreasing trend of HDL-C was significantly ameliorated in CM and CH groups.

In addition, the pathological structure of the livers was investigated in this study. Compared with the NC group, the pathological structure of the livers exhibited massive lipid accumulation and degeneration accompanied in the DC group when stained with H&E and Oil Red O. The levels of lipid accumulation and degeneration were significantly alleviated with the administration of CJE in a dose-dependent manner (Figure 4A). In addition, as the main organ for glucose production and storage, the liver plays a key role in maintaining glucose homeostasis by regulating glycogen synthesis and breakdown and gluconeogenesis pathways [32]. The results of PAS staining showed that the area of liver glycogen in the DC group decreased significantly, accounting for only 33.08% of the value for the NC group, but it showed a dose-dependent increase after CJE administration. Compared with the DC group, the area of liver glycogen in the PM, CL, CM, and CH groups increased by 170.94%, 52.93%, 108.84%, and 141.44%, respectively (Figure 4B). At the same time, the content of liver glycogen in T2DM mice was significantly increased in the CM (44.94%) and CH (76.06%) groups in comparison to the DC group (Figure 4C). This suggests that CJE could promote glycogen synthesis and storage in T2DM mice. The results for the liver pathological sections show a similar trend to blood glucose and lipid levels, indicating that the intervention with CJE could effectively ameliorate disturbances in glucose and lipid metabolism in T2DM mice.

### 3.5. CJE Affected the Diversity and Composition of the Intestinal Microbiota of T2DM Mice

**Figure 5:**
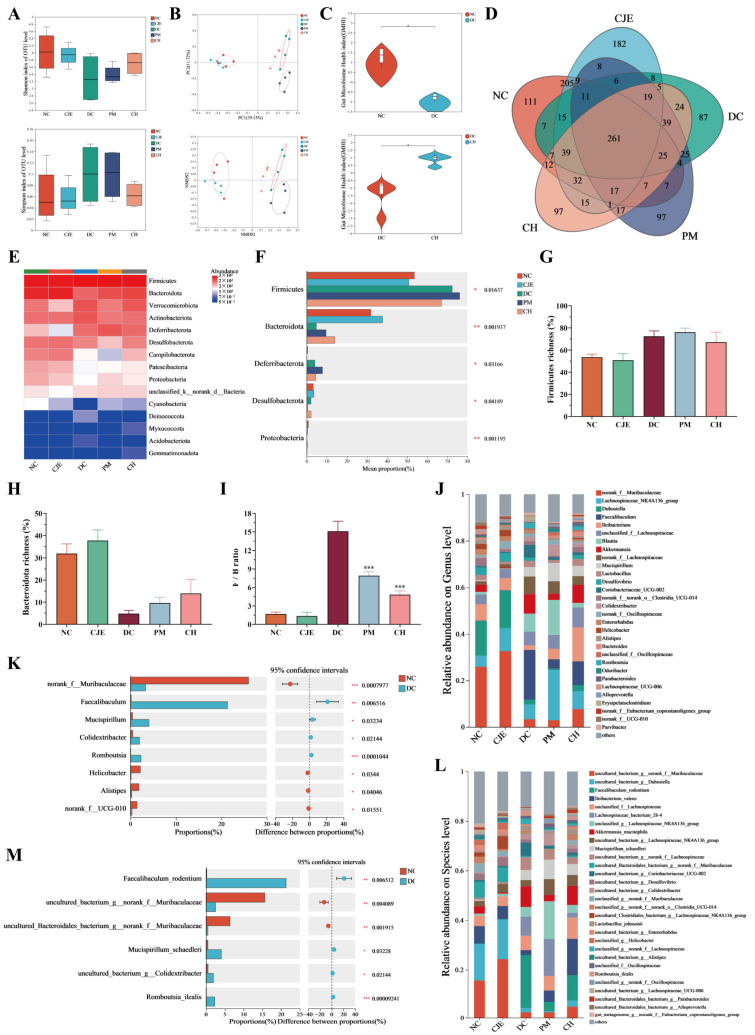
Effect of CJE on gut microbiota abundance and composition in T2DM mice (*n* = 4). (**A**) Alpha diversity analysis at the OTU level. (**B**) Beta diversity analysis by NMDS and PCoA methods at the OTU level. (**C**) Gut microbiome health index at the OTU level. (**D**) Venn diagram for OTUs. (**E**) Phylum-level distribution of gut microbiota. (**F**) Percent community abundance diagram at the phylum level in the five groups. (**G**) Firmicutes richness. (**H**) Bacteroidota richness. (**I**) F/B ratio. (**J**) Gut microbiota composition at the genus level in the five groups. (**K**) Percent community abundance diagram between the NC group and the DC group at the genus level. (**L**) Gut microbiota composition at the species level in each group. (**M**) Percent community abundance diagram between the NC group and DC group at the species level. Data are presented as mean ± SEM. * *p* < 0.05, ** *p* < 0.01, *** *p* < 0.001 vs. the DC group.

A recent study proved that disturbances in glucose and lipid metabolism in diabetic patients are related to the imbalance of intestinal homeostasis [33]. Therefore, the effect of CJE on gut microbiota composition and function was investigated in this study. The Simpson and Shannon indices of the DC group were not significantly different (*p* > 0.05) in comparison to the NC, CJE, and CH groups (Figure 5A). These results showed that the flora richness and community diversity of the gut microbiota were not significantly different in all the groups. The effects of CJE on beta diversity and the structural clustering of the gut microbiota were evaluated in this study (Figure S1). The results show that the microbial community structure and composition of the DC group and NC group were completely separated, indicating that the composition and structure of the gut microbiota in diabetic mice were significantly altered (Figure 5B). Moreover, the microbial community in the CH group showed high similarity to that in the NC group after the CJE intervention, suggesting that CJE can improve the structure of the gut microbiota in diabetic mice. The GMHI in the DC group was significantly lower than in the NC group, indicating that the dysbiosis of the gut microbiota existed in diabetic mice (Figure 5C). Compared with the DC group, the GMHI was significantly increased in diabetic mice receiving a high dose of the CJE treatment, which suggested that CJE could significantly improve the gut health index in diabetic mice. As shown in Figure 5D, the number of unique OTUs in the NC group, CJE group, DC group, PM group, and CH group was 111, 182, 877, 97, and 97, respectively (Figure 5D). These results indicate that the number of species in the gut microbiota in the T2DM mice was significantly lower than that in the healthy mice, and this was reversed by the CJE treatment. The taxonomic composition of the gut microbiota (GM) was analyzed at the phylum, genus, and species levels among the different groups. The gut microbiota of mice at the phylum level was mainly composed of *Firmicutes*, *Bacteroidota*, *Verrucomicrobia*, *Proteobacteria*, *Actinobacteria,* and *Deferribacterota* (Figure 5E). Compared with the NC group, the abundance of *Firmicutes* was increased (72.37%) and that of *Bacteroides* was significantly decreased (4.796%) in the DC group. (Figure 5F–H). Furthermore, the F/B ratio of the DC group was significantly higher than that of the NC group, which was reversed by the CJE treatment (Figure 5I). Compared with the NC group, the abundances of *Faecalibaculum*, *Mucispirillum*, *Colidextribacter*, and *Romboutsia* in the DC group were significantly increased and the abundances of norank_f__*Muribaculaceae*, *Helicobacter*, *Alistipes,* and *norank_f__UCG-010* were significantly reduced at the genus level (Figure 5J,K). As shown in Figure S2A–D, the abundances of Faecalibaculum, Coriobacteriaceae_UCG-002, and Romboutsia were significantly decreased and that of Ileibacterium was significantly increased in T2DM mice receiving the CJE treatment.

The abundances of Faecalibaculum_rodentium, Mucispirillum_schaedleri, uncultured_bacterium_g__Colidextribacter, and Romboutsia_ilealis in the DC group were significantly increased and the abundances of uncultured_bacterium_g__norank_f__Muribaculaceae and uncultured_Bacteroidales_bacterium_g_norank_f__Muribaculaceae were significantly reduced at the species level (Figure 5L,M). As shown in Figure S2E–H, the abundances of Faecalibaculum_rodentium, bacterium_g__Coriobacteriaceae_UCG-002, and Romboutsia_ilealis were significantly decreased and that of Ileibacterium_valens was significantly increased in T2DM mice receiving the CJE treatment. These results suggest that CJE can significantly ameliorate disturbances in the gut microbiota in diabetic mice.

### 3.6. CJE Improved Metabolic Homeostasis in T2DM Mice by Mediating the Intestinal Microbiota

**Figure 6:**
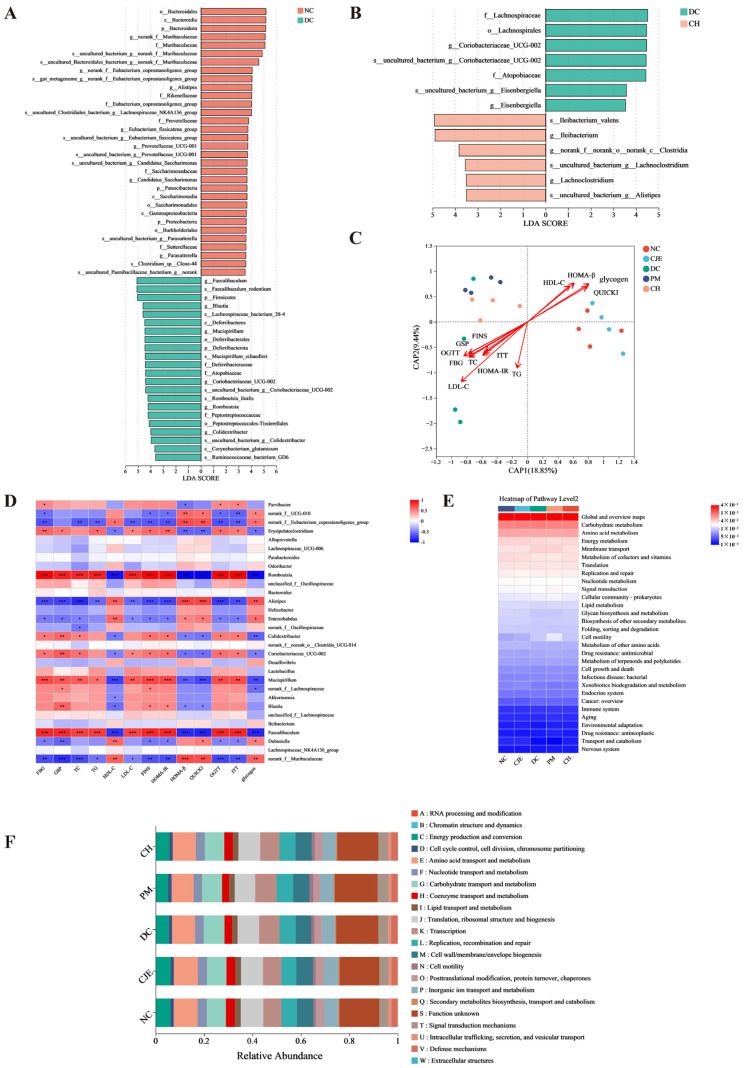
Association of intestinal microbiota composition and function under the CJE intervention in T2DM mice and prediction of metabolic pathways. (**A**) LEfSe analysis of intestinal flora between the NC group and the DC group from the phylum level to the species level. (**B**) LEfSe analysis between the DC group and the CH group. (**C**) Distance-based redundancy analysis at the genus level. (**D**) Pearson correlation analysis between the top 30 microbial genera in the intestinal flora and related metrics of glucose metabolism homeostasis. (**E**) COG function classification. (**F**) Average abundance of KEGG pathways for the five groups at level 2. * *p* < 0.05, ** *p* < 0.01, *** *p* < 0.001.

LEfSe analysis was used to compare the relative abundance of species in the gut microbiota of different groups, and the bacteria displaying significant differences between groups were identified at the phylum, genus, and species levels. A taxon was considered a marker species when *p* < 0.05 and when the Linear Discriminant Analysis (LDA) score ≥ 3.5. The results show that *Bacteroidetes* and *Firmicutes* were the most enriched key differential bacteria between the NC group and DC group (Figure 6A), which was consistent with the analysis at the phylum level (Figure 5E,F). As shown in Figure 5J–M, compared with the NC group, the key differences in the bacterial flora of *Faecalibaculum*, *Mucispirillum*, *Colidextribacter*, and *Romboutsia* were significantly increased in the DC group. The key differences in bacterial flora of *Ileibacterium*, *Clostridia*, *Lachnoclostridium*, and *Alistipes* were significantly increased in T2DM mice after the CJE intervention (Figure 6B). These results suggest that CJE might alleviate disturbances in glucose and lipid metabolism in T2DM mice by increasing the abundance of *Alistipes* and reducing the abundance of *Faecalibaculum*, *Coriobacteriaceae*, and *Romboutsia*.

In order to explore the relationship between the body homeostasis index and the gut microbiota in T2DM mice under the intervention with CJE, Db-RDA (distance-based redundancy) analysis was performed based on the distance matrix. Db-RDA analysis showed that the CJE intervention was negatively correlated with the blood glucose homeostasis indexes FBG, GSP, TC, TG, LDL-C, FINS, and HOMA-IR and positively correlated with HDL-C, HOMA-β, and QUICKI (Figure 6C). As shown in Figure 6D, the abundance of *Alistipes* was negatively correlated with FBG, GSP, TC, TG, LDL-C, FINS, and HOMA-β and positively correlated with HDL-C, HOMA-β, QUICKI, and hepatic glycogen. The dominant bacteria of *Faecalibaculum*, *Coriobacteriaceae,* and *Romboutsia* enriched in T2DM mice showed the opposite correlation with various homeostasis indicators, and the abundances of these three bacteria were significantly reduced after the CJE intervention. These results indicate that CJE interfered with the gut microbiota of *Alistipes*, *Faecalibaculum*, *Coriobacteriaceae*, and *Romboutsia*, which are closely related to glucose and lipid metabolism homeostasis in T2DM mice. Combined with the analysis of 16S rRNA sequencing data from the eggNOG database, it was observed that the intestinal flora of each group was involved in a variety of functions, mainly focused on energy production and conversion, gene expression, and the transport and metabolism of carbohydrates, amino acids, lipids, and coenzymes (Figure 6E). The metabolic pathways of the sequencing data were predicted based on the Kyoto Encyclopedia of Genes and Genomes (KEGG) database. The results show that on the second level, carbohydrate, amino acid, and energy metabolism pathways, as well as membrane transport pathways, dominated the functions of the gut microbiota (Figure 6F).

### 3.7. CJE Regulated Hypoglycemic Signaling Pathways in the Liver of T2DM Mice

**Figure 7:**
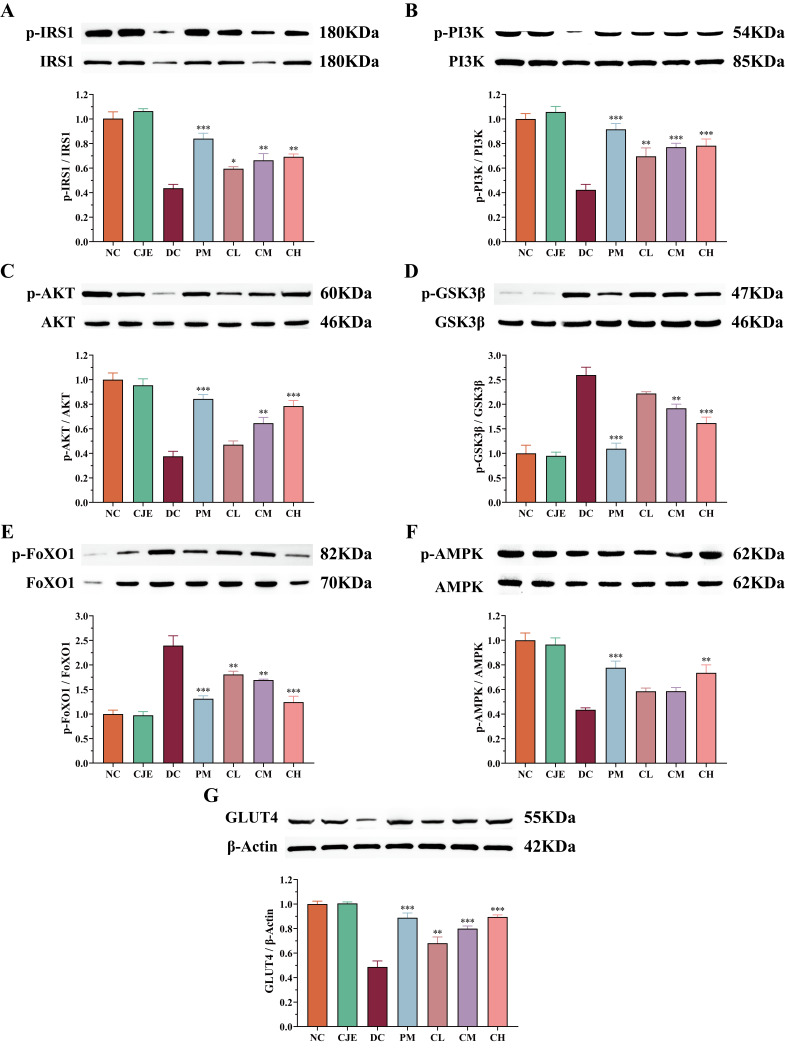
Effects of CJE administration on the IRS1-PI3k-AKT-FoxO1/GSK 3β signaling pathway and the AMPK signaling pathway in the liver of T2DM mice. (**A**) p-IRS1 and IRS. (**B**) p-PI3K and PI3K. (**C**) p-Akt and Akt. (**D**) p-FoxO1 and FoxO1. (**E**) p-GSK-3β and GSK-3β. (**F**) p-AMPK and AMPK. (**G**) GLUT4. Data are presented as mean ± SEM. * *p* < 0.05, ** *p* < 0.01, *** *p* < 0.001 vs. the DC group.

The expression levels of marker proteins in key pathways of glucose metabolism were determined for elucidating the mechanisms of CJE regulating glucose and lipid metabolism disruptions by Western blotting. The results showed that the phosphorylation levels of IRS1, PI3K, and Akt in the DC group were lower, accounting for only 43.36%, 42.77%, and 37.60% of the NC group (Figure 7A–C). Compared with the DC group, the phosphorylation levels of IRS1 (58.62%), PI3K (85.05%), and Akt (108.76%) were significantly increased in T2DM mice receiving a high dose of the CJE treatment (Figure 7A–C). GSK-3β and FoxO1 are negatively regulated genes related to glucose metabolism. Activation of the PI3K-Akt pathway further inhibits the phosphorylation of GSK-3β and FoxO1 in healthy mice [34]. As the phosphorylation of AKT, an indirect substrate of the insulin signaling pathway, was inhibited in T2DM mice, the phosphorylation levels of GSK-3β and FoxO1 in the livers of the DC group were significantly higher than those for the NC group (Figure 7D,E). However, this change was significantly reversed by CJE administration. These results suggest that CJE could activate the phosphorylation of the IRS1-PI3K-Akt pathway and inhibit the phosphorylation of FoxO1/GSK-3β to promote hepatic gluconeogenesis and glycogen synthesis, thus alleviating glucose metabolism disruptions and insulin resistance in T2DM mice. AMPK, as a key regulator of the energy balance by increasing glucose uptake and inhibiting intracellular glucose production [35], plays an important role in the prevention and treatment of diabetes [36]. The phosphorylation level of AMPK in the livers of the DC group mice was significantly lower than that for the control group, and this was reversed by the CJE treatment (Figure 7F). The expression level of GLUT4 protein directly affects the body’s glucose utilization [37]. When GLUT4 is involved in an insulin-independent glucose uptake pathway, its expression level is regulated by AMPK [38]. The up-regulation of AMPK phosphorylation activated the expression of GLUT4, and the expression level of GLUT4 in T2DM mice was significantly increased after the CJE intervention (Figure 7G). Compared with the DC group, the expression of GLUT4 in the high-dose group was up-regulated by 83.84%. This suggests that CJE can up-regulate GLUT4 expression by activating AMPK phosphorylation, thereby increasing glucose transport and uptake capacity in T2DM mice.

## 4. Discussion

In this study, the diabetic mouse model induced by an HFD and STZ was used as the experimental object to explore the therapeutic effect and mechanisms of CJE’s impact on T2DM mice. Firstly, the results of basal physiological indicators preliminarily indicate that CJE has the potential to treat T2DM (Figure 1). Moreover, the results of blood glucose monitoring showed that CJE has significant hypoglycemic activity and can reduce the fasting blood glucose level in T2DM mice in a dose-dependent manner and that the intervention with a high dose of CJE can achieve a similar therapeutic effect as metformin (Figure 2A). At the same time, it was found that the CJE intervention with 6 weeks of a high dose had no significant effect on FBG levels in normal healthy mice, indicating that CJE had no potential risk of inducing hypoglycemia. GSP is the product of a slow, non-enzymatic glycation reaction of various proteins in the serum with glucose, which reflects the average blood glucose level over a 3-week period [28]. And, the level of GSP was consistent with the changing trend of FBG, which further confirmed that CJE could reduce hyperglycemia caused by T2DM.

Type 2 diabetes mellitus is characterized by insulin resistance and pancreatic β-cell dysfunction [39]. And, as an important organ involved in the body’s digestive and glucose metabolism pathways, the repair of pancreatic functional damage has been proven to be an effective way to treat T2DM [40]. The pathological morphology of the pancreas, such as focal necrosis and inflammatory infiltration, was significantly improved in T2DM mice after the CJE intervention (Figure 3). In addition, insulin is secreted by pancreatic β-cells and plays an important role in maintaining the stability of blood glucose levels. The evaluation of the number and morphological function of β-cells has been recognized as the hallmarks of diabetes [41]. The CJE intervention restored the morphology and function of islet β-cells, which increased insulin sensitivity in T2DM mice. Moreover, the increase in insulin sensitivity further enhanced the body’s ability to regulate glucose metabolism, thus maintaining blood glucose homeostasis. And, this is also consistent with the results of the OGTT and ITT experiments.

A previous study confirmed that disturbances in lipid metabolism are closely related to fatty liver disease, insulin resistance, and hyperglycemia [42]. Meanwhile, as one of the important target organs of insulin, the liver plays an important role in the balance between glycogenolysis and gluconeogenesis [31]. CJE significantly increased the area and content of liver glycogen in T2DM mice and restored glycogen storage. This leads to further speculation that insulin-mediated metabolic pathways may exist in the liver to regulate glucose metabolism. Moreover, as an important organ in lipid synthesis and metabolism, the liver plays a dominant role in maintaining lipid homeostasis. It has been reported that T2DM is closely related to liver inflammatory infiltration, steatosis, and other lesions [43]. Lipid metabolism pathways are negatively affected in T2DM mice due to impaired liver function. And, the CJE intervention significantly ameliorated the abnormal morphology and structure of hepatocytes in T2DM mice, effectively improving pathological changes, such as lipid accumulation and steatosis. Previous studies have shown that abnormal lipid deposition in the liver of T2DM is closely related to insulin resistance, which is usually accompanied by different degrees of lipid metabolism disturbances in the body [42,44]. The results of this study suggested that the increase in insulin sensitivity and the improvement in insulin resistance by CJE ameliorate lipid metabolism disruptions, which further inhibit the abnormal changes in lipid profile indices in T2DM mice.

The gut microbiota plays an important role in a variety of metabolic pathways. Emerging research has suggested that part of the intestinal flora plays a key role in the development of T2DM via metabolites of secondary bile acids and short-chain fatty acids increasing insulin resistance [45,46], and the regulation of the gut microbiota can ameliorate obesity and glucose and lipid metabolism disruptions caused by T2DM [13,47]. At the phylum level, the CJE intervention reversed the increase in *Firmicutes* abundance and the decrease in *Bacteroides* abundance in T2DM mice, significantly reducing the F/B ratio. This efficacy has been confirmed for a variety of polyphenols [38,48,49,50]. The increase in the abundance of *Faecalibaculum* and *Coriobacteriaceae* generally aggravates insulin resistance induced by a high-fat diet by promoting lipid accumulation [51,52]. The abundance of *Faecalibaculum* and *Coriobacteriaceae* significantly decreased after the CJE intervention, thereby improving lipid accumulation-mediated insulin resistance in T2DM mice. Previous studies have shown that the abundance of Romboutsia significantly increased with a high-fat diet [53]. Concurrently, the production of short-chain fatty acids significantly decreased [54]. In addition, LPS exposure tends to be attributed to an increased level of Gram-negative bacteria (such as Romboutsia) development in the lumen of intestines in high-fat diet mice [55]. The accumulation of LPS disrupted intestinal barrier function, causing excess LPS to be absorbed into the intestinal lumen and potentially circulate in critical ways to the islets and liver, leading to insulin resistance [56]. The abundance of *Romboutsia* was significantly reduced under the CJE intervention, which alleviated liver lipid accumulation and pathological status and further improved the disturbances of glucose and lipid metabolism in T2DM mice [57]. Meanwhile, our results demonstrated a positive correlation between *Romboutsia* abundance and FBG levels, which is consistent with the results of Mei et al. [58]. In contrast, an increase in the abundance of *Alistipes* is beneficial for alleviating insulin resistance, which has been demonstrated in the study by Takeuchi et al. [59]. The abundance of *Alistipes* increased significantly under the intervention of CJE. In addition, SCFAs produced by *Alistipes* can maintain energy homeostasis and increase the insulin sensitivity of the host. This alleviates insulin resistance in T2DM mice, thereby improving liver regulation of glucose and lipid metabolism. This way of T2DM mediated by *Alistipes* has been confirmed in other flavonoids [60]. In summary, CJE ameliorated insulin resistance by reducing the abundance of *Faecalibaculum*, *Coriobacteriaceae*, and *Romboutsia* and increasing the abundance of *Alistipes*, thereby mediating the regulation of glucose and lipid metabolism in the liver.

The PI3K/Akt pathway regulates metabolic processes, such as hepatic gluconeogenesis, glycolysis, and glycogen synthesis [61,62], plays a key role in insulin signaling, and is essential for maintaining the homeostasis of glucose and lipid metabolism [63]. As the mediator of most of the metabolic effects of insulin receptor (INSR) activation, the phosphorylation of IRS-1 is required for the development of insulin sensitivity, thereby activating PI3K [64,65]. PI3K is an intracellular signaling enzyme with two subunits, p85 and p110, whose effects on hepatic glucose and lipid metabolism are mediated through Akt [66], and the recruitment of PI3K significantly promotes the phosphorylation of Akt [67]. Akt, a downstream target of PI3K, is an important multi-functional protein kinase [68]. Activated Akt can inhibit the protein expression of GSK3β and promote the synthesis of liver glycogen [69]. As a transcription factor involved in G6P and PEPCK activation, FoxO1 is responsible for the regulation of hepatic gluconeogenesis [70]. However, activation of the PI3K/Akt pathway inhibits the phosphorylation of FoxO1 [71]. In this study, CJE was able to significantly activate the phosphorylation of RS1-PI3K-Akt and inhibit the phosphorylation of GSK 3β and FoxO1 in T2DM mice. AMPK is a key regulator in maintaining cellular energy homeostasis [72]. CJE significantly up-regulated AMPK phosphorylation in T2DM mice (Figure 7F). It has been shown that AMPK is closely related to the PI3K/AKT signaling pathway and that activation of AMPK can improve insulin resistance by promoting the oxidative consumption of fatty acids in the liver and reducing ectopic accumulation of lipids [73,74]. Numerous studies have shown that hepatic gluconeogenesis and glycogen synthesis are also improved through the insulin-independent AMPK pathway [75]. GLUT4 is the major protein responsible for glucose transport and plays an important role in glucose uptake and metabolism [76]. The activation of PI3K/AKT can up-regulate the expression of GLUT4 and ameliorate insulin resistance, which is an insulin-mediated process [77,78]. Phosphorylated AMPK activates GLUT4 expression, which in turn promotes glucose transport to the cell membrane [79]. CJE could inhibit gluconeogenesis and promote glycogen synthesis through the insulin-mediated IRS1-PI3K-Akt-FoxO1/GSK 3β pathway and increase glucose transport through the AMPK-GLUT4 pathway, thereby regulating glucose and lipid metabolism disorders in T2DM mice.

However, there are still some limitations to this study, such as the hypoglycemic activity, which was not analyzed for the monomeric flavonoids in extracts from chuju, and the reliance on 16S rRNA sequencing for gut microbiota analysis, which lacks species-level resolution and functional insights. Accordingly, in subsequent experiments, we will investigate the hypoglycemic activity of monomer flavonoids, which are abundant in chrysanthemum extracts. Moreover, metagenomic next-generation sequencing will be further used to probe for key strains that regulate glycolipid metabolism.

## 5. Conclusions

In this study, we found that the CJE can significantly improve hyperglycemia in T2DM mice. CJE can significantly improve glucose tolerance and insulin sensitivity in T2DM mice by reducing liver lipid accumulation, promoting the regeneration and recovery of islet β-cells, reducing insulin resistance, and thus ameliorating glucose and lipid metabolism disturbances in T2DM mice. Simultaneously, CJE has the ability to alleviate disturbances in the intestinal flora and can improve the structure of the gut microbiota in T2DM mice, bringing it closer to that of healthy mice. CJE can down-regulate the phosphorylation of FoXO1 and GSK3β by activating the PI3K-AKT signaling pathway, thereby promoting glycogen synthesis and inhibiting gluconeogenesis. In addition, CJE up-regulates the expression of GLUT4 by activating the AMPK pathway, thereby promoting glucose transport. These results provide a theoretical basis for the high-value utilization of GJE and also present novel perspectives for the development of preventive methods and future clinical investigation for T2DM.

## Figures and Tables

**Table 1:** The contents of the main flavonoids in CJE.

Flavonoids	Content (μg/g)
Apigenin 6, 8-digalactoside	77,245.92831
Apigenin 6-C-glucoside 8-C-arabinoside	60,064.4289
Luteolin-4′-O-glucoside	54,672.57871
Isoshaftoside	34,516.45459
Scutellarin	24,582.14705
Quercetin 3-O-malonylglucoside	24,113.02598
Chrysoeriol 7-O-glucoside	16,414.19393
Quercetin-3,4′-O-di-beta-glucoside	14,663.34312
Luteolin 6-C-glucoside 8-C-arabinoside	13,151.14052
Homoorientin	11,983.51217

## Data Availability

The raw data supporting the conclusions of this article will be made available by the authors upon request.

## Supplementary Materials

The following supporting information can be downloaded at https://www.mdpi.com/article/10.3390/foods14050765/s1, Figure S1: Hierarchical clustering tree at the OTU level in T2DM mice; Figure S2: Relative abundance of microbial species of T2DM mice feces. (A-D) at the genu level, (E-H) at the specie level. Data are presented as mean ± SEM. * *p* < 0.05, ** *p* < 0.01, *** *p* < 0.001 vs. the DC group.

## Abbreviations

AUC	area under curve
BW	body weight
CH	diabetic with high-dose CJE group
CJE	extracts from chuju
CL	diabetic with low-dose CJE group
CM	diabetic with medium-dose CJE group
COG	Clusters of Orthologous Groups
Db-RDA	distance-based redundancy
DC	diabetic control group
SCFAs	short-chain fatty acids
FBG	fasting blood glucose
FINS	fasting serum insulin
GM	gut microbiota
GLUT4	Glucose transporter type 4
GMHI	gut microbiome health index
GSP	glycosylated serum protein
H&E	hematoxylin-eosin
HDL-C	high-density lipoprotein cholesterol
HFD	high-fat diet
HOMA-IR	Homeostasis model assessment of insulin resistance
HOMA-β	Homeostasis model assessment of β-cell function
INSR	insulin receptor
ITT	Insulin tolerance test
KEGG	Kyoto Encyclopedia of Genes and Genomes
LDA	Linear Discriminant Analysis
LDL-C	low-density lipoprotein cholesterol
NC	normal control group
NMDS	non-metric multidimensional scaling
OGTT	oral glucose tolerance test
OTU	Operational Taxonomic Units
PAS	Periodic Acid-Schiff
PCoA	principal co-ordinates
p-Akt	phosphor-Protein Kinase B
p-AMPK	Adenosine 5′-monophosphate (AMP)-activated protein kinase
PAS	Periodic Acid-Schiff
p-FoxO1	phosphor-Forkhead Box O1
p-GSK3β	phosphor-Glycogen Synthase Kinase 3 Beta
p-IRS-1	phosphor-Insulin Receptor Substrate 1
PM	positive control group
p-PI3K	phosphor-Phosphatidylinositide 3-kinases
PVDF	polyvinylidene fluoride
QUICKI	quantitative insulin sensitivity check index
SCFAs	short-chain fatty acids
SDS-PAGE	sodium dodecyl sulfate-polyacrylamide gel electrophoresis
STZ	streptozotocin
T2DM	type 2 diabetes mellitus
TC	total cholesterol
TG	total triglyceride
UPLC-QTOF	Ultra-high performance liquid chromatography-quadrupole time-of-flight mass spectrometry
WAT	white adipose tissue
