# Supplementary material for: Flavonoid-Rich Extracts from Chuju (Asteraceae Chrysanthemum L.) Alleviate the Disturbance of Glycolipid Metabolism on Type 2 Diabetic Mice via Modulating the Gut Microbiota
Source: Foods. 2025 Feb 24;14(5):765. doi: 10.3390/foods14050765 (PMC11898795; doi:10.3390/foods14050765)
Supplement: Supplementary file 1 [file foods-14-00765-s001.zip › foods-3430646-supplementary.pdf]

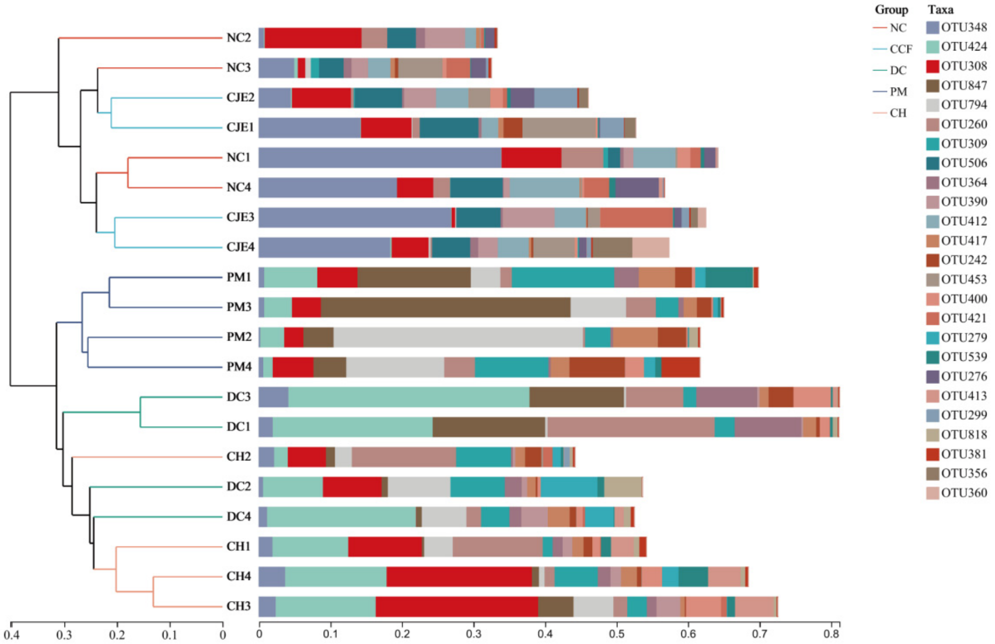

Figure S1. Hierarchical clustering tree at the OTU level in T2DM mice.

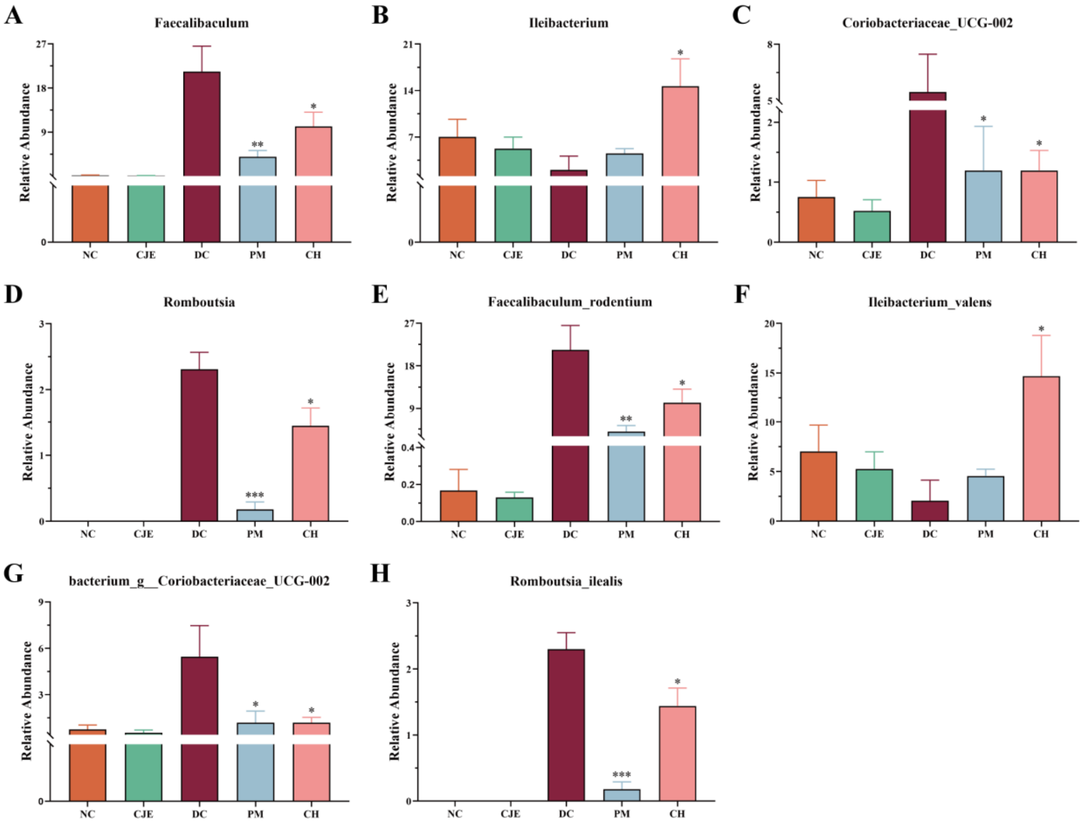

Figure S2. Relative abundance of microbial species of T2DM mice feces. (A-D) at the genu level, (E-H) at the specie level. Data are presented as mean ± SEM. *$P < 0.05$, **$P < 0.01$, ***$P < 0.001$ versus the DC group.
